# Validation and Search of the Ideal Cut-Off of the Sysmex UF-1000i^®^ Flow Cytometer for the Diagnosis of Urinary Tract Infection in a Tertiary Hospital in Spain

**DOI:** 10.3389/fmed.2018.00092

**Published:** 2018-04-09

**Authors:** María I. Millán-Lou, Juan M. García-Lechuz, María A. Ruiz-Andrés, Concepción López, María J. Aldea, María J. Revillo, Antonio Rezusta

**Affiliations:** ^1^Servicio de Microbiología, Hospital Universitario Miguel-Servet, Zaragoza, Spain; ^2^Instituto de Investigación Sanitaria Aragón (IIS Aragón), Zaragoza, Spain; ^3^Universidad de Zaragoza, Zaragoza, Spain

**Keywords:** UF-1000i, validation, cut-off, flow cytometer, urinary tract infection, bacteria, leukocytes

## Abstract

Urinary tract infections (UTI) are one of the most prevalent infections. A rapid and reliable screening method is useful to screen out negative samples. The objective of this study was to validate the Sysmex flow cytometer UF-1000i by evaluating its accuracy, linearity and carry-over; and define an optimal cut-off value to be used in routine practice in our hospital. For the validation of the UF-1000i cytometer, precision, linearity and carry-over were studied in samples with different counts of bacteria, leukocytes and erythrocytes. Between March and June 2016, urine samples were tested in the Clinical Microbiology Laboratory at University Miguel Servet Hospital, in Spain. Samples were analyzed with the Sysmex UF-1000i cytometer, and cultured. Growth of ≥10^5^ CFUs/mL was considered positive. The validation study reveals that the precision in all the variables is acceptable; that there is a good linearity in the dilutions performed, obtaining values almost identical to those theoretically expected; and for the carry-over has practically null values. A total of 1,220 urine specimens were included, of which 213 (17.4%) were culture positive. The optimal cut-off point of the bacteria–leukocyte combination was 138.8 bacteria or 119.8 leukocytes with an S and E of 95.3 and 70.4%, respectively. The UF-1000i cytometer is a valuable method to screen urine samples to effectively rule out UTI and, may contribute to the reduction of unnecessary urine cultures.

## Introduction

Urinary tract infections (UTIs) are the most common bacterial infections (1–3). However, 70–80% of cultures are negative (4–6). *Escherichia coli* is the most prevalent uropathogen (7) and is responsible for approximately 80% of uncomplicated community-acquired UTI (8).

Quantitative urine culture and identification are still the “gold standard” laboratory procedures for definitive diagnosis of urinary tract infections, but it is labour-intensive, time-consuming and does not provide same-day results (3, 9–15). Therefore, a rapid and reliable screening method is useful to screen out negative samples, in order to reduce the overall turnaround time of analyses, workload and costs (12, 16). To prevent positive urine samples from erroneously being classified as negative and not being cultured, a high sensitivity and negative predictive value (NPV) are prerequisites for a screening method (14, 17). The European Urinalysis Guidelines recommend an analytical sensitivity >90–95% to detect asymptomatic bacteriuria at 10^8^ colony-forming bacteria/litre (CFB/L), equivalent to 10^5^ colony-forming unit (CFU)/millilitre (mL), by a rapid non-culture method, with a confirmatory culture of positive cases (18).

Flow cytometry analysis has long been recognized as capable of identifying bacteria (19) and the Sysmex UF-1000i automated urine particle flow cytometer has been developed to standardize urine sediment analysis. This automated analyser rapidly quantifies urine particles, including white blood cells (WBCs), bacteria, red blood cells (RBCs), and casts by scattering and fluorescence (after staining) (12). This instrument has a separate measurement channel for bacteria that improves the specificity for counting bacterial organisms (13, 15). Previous studies have demonstrated that this system has good precision with low interference, low carryover contamination (11). The use of urine flow cytometry and the introduction of a cut-off value, which determines if urine is subsequently cultured or not, can reduce the number of cultures (12).

The aim of this study was to validate the Sysmex UF-1000i flow cytometer by the evaluation of its precision, linear estimation of results and carry-over contamination rate, the comparison of its performance to bacterial culture and define a cut-off value to be used in routine practice in our hospital.

## Materials and Methods

### Testing of Precision, Linearity and Carry-Over of the UF-1000i

#### Precision

The within-run precision was determined by measuring the RBCs, WBCs, casts, bacteria, and epithelial cells in five samples. Each sample was continuously examined 10 times. The results of the examinations were recorded and analysed using the coefficient of variation (CV). The point where the CV exceeded 40% was arbitrarily defined as the lower limit of quantification.

#### Linearity

Three high-value urine samples with values close to the expected upper limit (RBCs, 10,967/μL; WBCs, 5,100/μL) were selected and diluted at ratios of 1:4, 1:16, 1:64, 1:256 and 1:1,024 in negative urine (all indexes near 0). The measured values were compared with the theoretical values, and the correlation coefficient was used to estimate Pearson linear correlation.

#### Carry-Over

Carry-over was determined by measuring a sample with high-value counts in triplicate (H1, H2, H3), followed by three consecutive measurements of a sample with low values (L1, L2, L3). Carry-over was calculated as follows: [(L1 − L3)/(H3 − L3)] × 100%. It was established that there is no substantial carry-over when this is less than 1%.

### Collection of Urine Specimens for Culture

Between March and June 2016, a total of 1,220 urine samples from inpatients and outpatients were tested in the Clinical Microbiology Laboratory at University Miguel Servet Hospital, in Spain. Sample size was determined by PASS v13 (NCSS Statistical Software) based in the Lin and Fine method, using a 95% of sensitivity and a precision of 5% for the expected UTI prevalence in our population. This study was approved by the local ethics committee (Comité de Ética de la Investigación de la Comunidad de Aragón (CEICA), reference number: 07/2016).

All urine specimens included in this study were tested by culture and UF-1000i cytometer analyser within 24 h of collection from Tuesday to Friday each week. Samples were excluded from analysis if excessive mucus, gross haemolysis or pyuria were noted upon visual inspection, or if inadequate sample volume (<8 mL) was available to prevent blockage of the instrument or interference during the measurement.

### Urine Culture

Prior to flow cytometry, the urine specimens were cultured using a WASP^®^DT: Walk*-*Away Specimen Processor (Copan Diagnostics, Murrieta, CA, USA) on Brilliance UTI agar (Oxoid Ltd., Basingstoke, United Kingdom). Cultures were incubated at 35°C for 18–24 h. Bacterial counts were expressed as the number of colony-forming units per millilitre. Growth of ≥10^5^ CFUs/mL was considered positive. Grown colonies were identified by MALDI-TOF (MALDI Microflex LT, Bruker Daltonics, Bremen, Germany). If there were three or more types of colonies without a dominant species, the urine culture was considered as contaminated but classified as negative and not subjected to the identification procedure.

### Urinalysis

After culture, the urine specimens were analysed in the Sysmex UF-1000i flow cytometer. The UF-1000i is a urine flow cytometer that uses a diode laser to quantify sediment in two analytic channels, and a fluorescent dye which stains DNA. One channel analyses only the microbial contents of the urine, while the other analyses RBCs, WBCs, casts and other non-microbial sediment. The staining agent is a fluorescent polymethine dye that binds to DNA. After staining, the particles are transported to a flow cell and are irradiated by a semiconducting laser (λ 635 nm). Forward scatter, side scatter, and fluorescence intensities of the individual particles are detected and information about particle size and structure is shown, which drives to identify and count the particles. The results are presented in histograms and scattergrams.

### Data Analysis

Statistical analysis was carried out by NCSS v10 and StatR v3.3.1. The correlation coefficient was used to estimate the linear correlation of theoretical vs. actual counts of RBCs and WBCs as measured by the UF-1000i. A ROC curve to determine the best cut-off values for bacteria and WBCs were calculated. Positive predictive value (PPV), NPV and accuracy rate at the best cut-off values for bacteria and WBCs were also calculated considering the urine culture as the reference.

## Results

### Sysmex UF-1000i Technical Validation

#### Precision

Precision of the UF-1000i in identifying the five formed components in urine is provided in Table 1. The intervals of the CV was presented in each component: RBCs 7.5–13.5% (19.34–10.84/μL); WBCs 13.5–31.8% (12.57–1.14/μL); bacteria high-value counts 2.8–29.7% (8.2–632.8/μL); bacteria low value counts 82.4–98.8% (1.6–2.0/μL); epithelial cell 9.0–34.6% (22.16–1.11/μL); and casts 60.4–152.9% (0.29–0.12/μL). The precision in all variables is acceptable, with values below the limit of 40%, except in CAST and low value counts of bacteria. In these cases, the average number of cylinders and bacteria in urine of all samples analysed is close to 0, which leads to a mathematical artefact with an excessively high CV.

**Table 1:** Precision of the UF-1000i.

		Sample 1	Sample 2	Sample 3	Sample 4	Sample 5
RBC	Average	12.43	22.57	19.34	10.84	6.25
	CV (%)	10.2	13.3	7.5	13.5	10.1
WBC	Average	1.46	5.65	1.14	6.83	12.57
	CV (%)	24.6	18.5	31.8	18.9	13.5
EC	Average	1.11	22.16	1.11	7.14	10.4
	CV (%)	27.7	9.0	34.6	14.1	13.4
CAST	Average	0.121	0.402	0.052	0.295	0.416
	CV (%)	152.9	74.3	129.1	60.4	67.6
BACTERIA	Average	1.62	8.18	1.98	46.71	632.83
	CV (%)	98.8	29.7	82.4	18.6	2.8

*RBC, red blood cells; WBC, white blood cells; EC, epithelial cells; CV, coefficient of variation*.

#### Linearity

**Figure 1:**
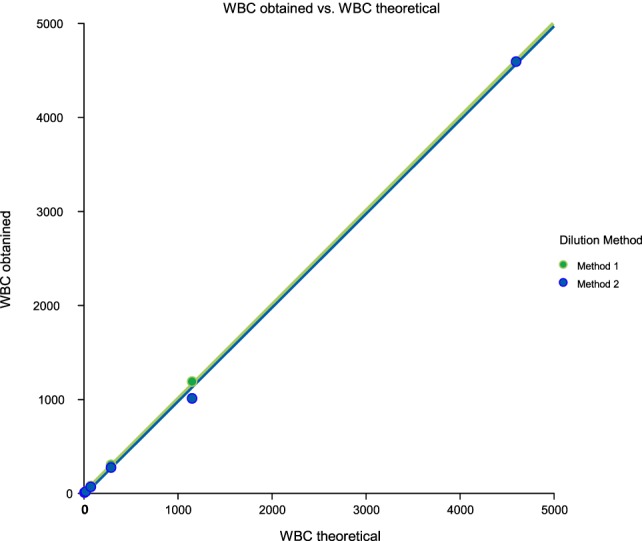
Scatter diagram of obtained and theoretical white blood cell (WBC) in sample 1.

**Figure 2:**
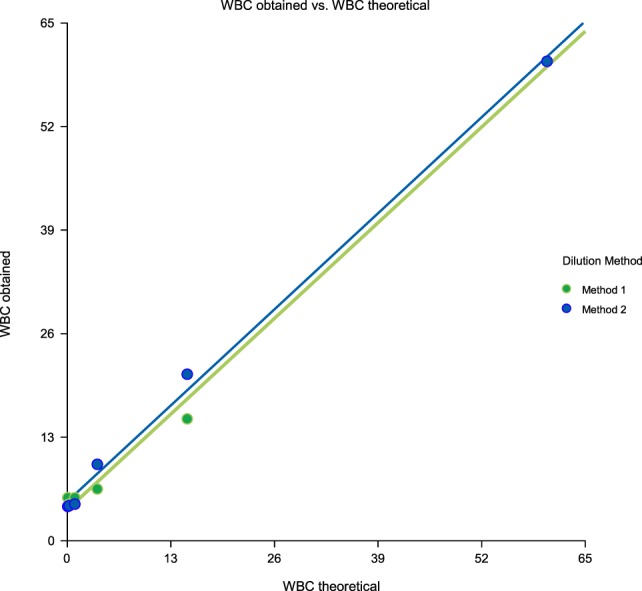
Scatter diagram of obtained and theoretical white blood cell (WBC) in sample 2.

**Figure 3:**
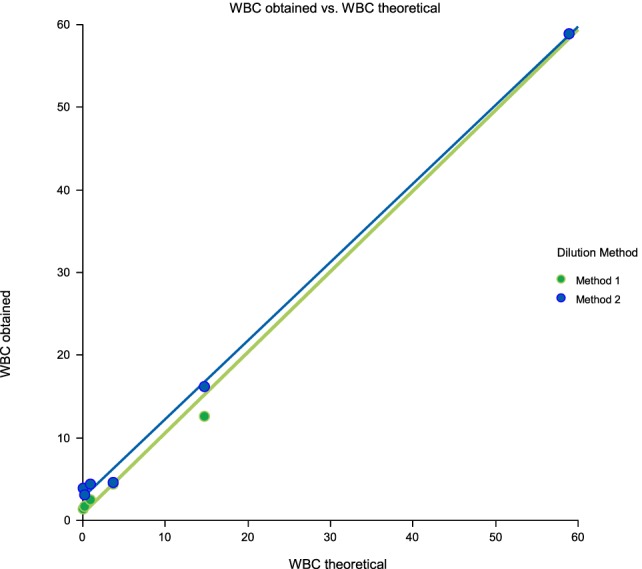
Scatter diagram of obtained and theoretical white blood cell (WBC) in sample 3.

**Figure 4:**
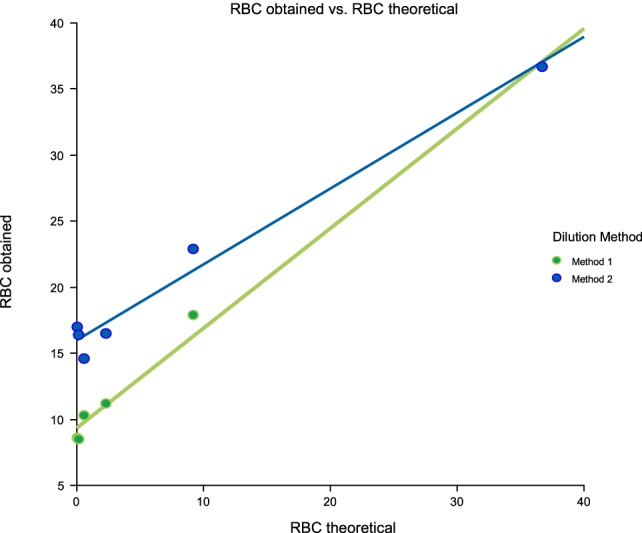
Scatter diagram of obtained and theoretical RBC in sample 1.

**Figure 5:**
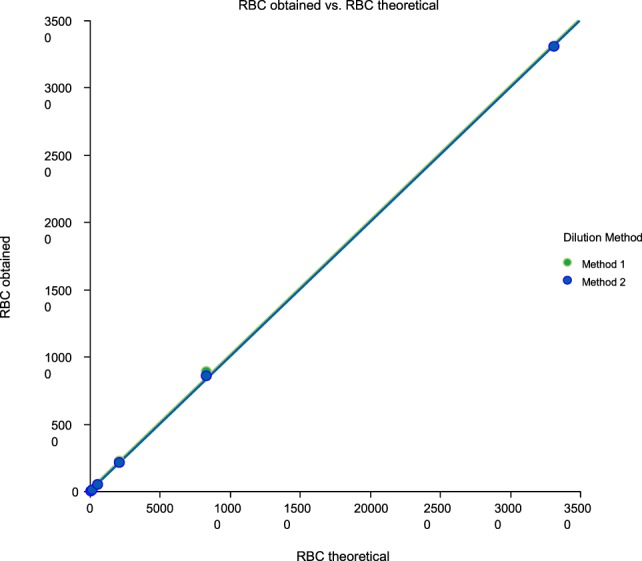
Scatter diagram of obtained and theoretical RBC in sample 2.

**Figure 6:**
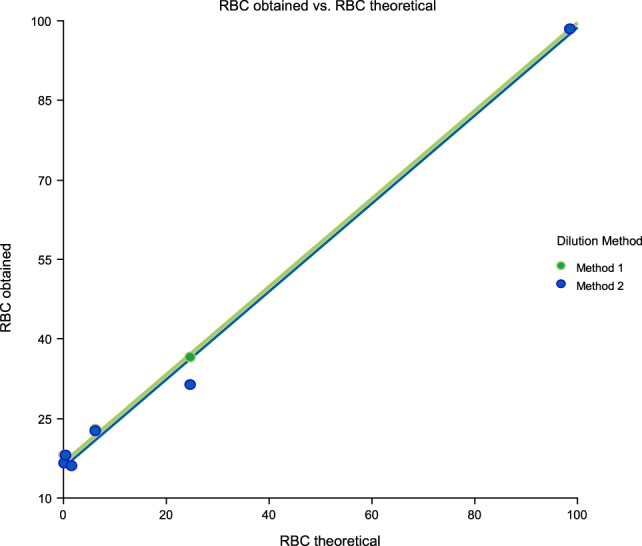
Scatter diagram of obtained and theoretical RBC in sample 3.

**Figure 7:**
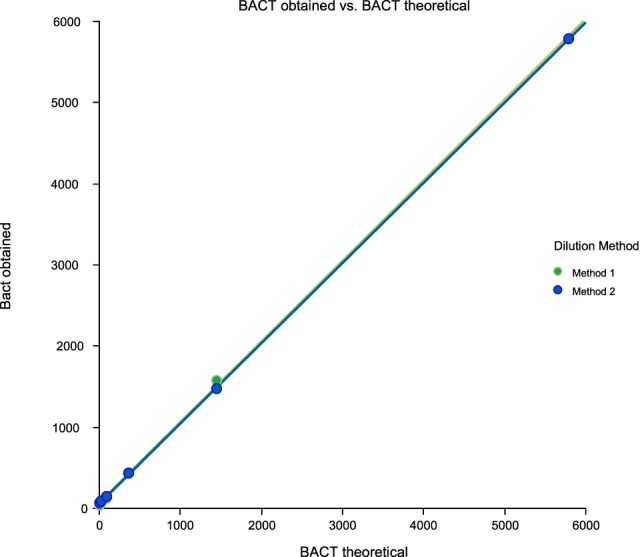
Scatter diagram of obtained and theoretical bacteria in sample 1.

**Figure 8:**
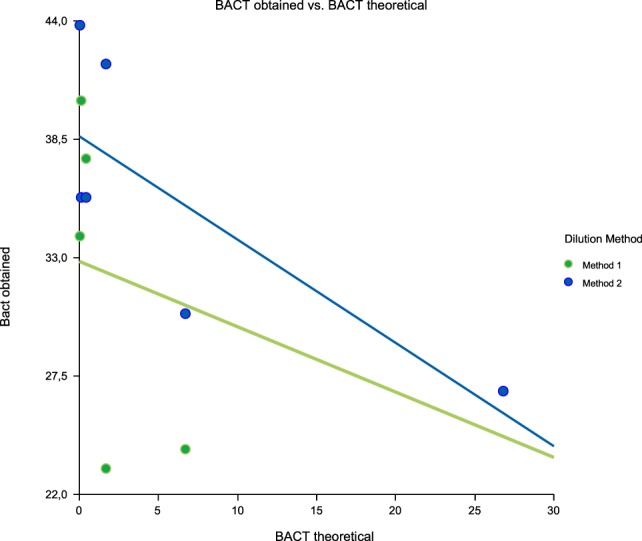
Scatter diagram of obtained and theoretical bacteria in sample 2.

**Figure 9:**
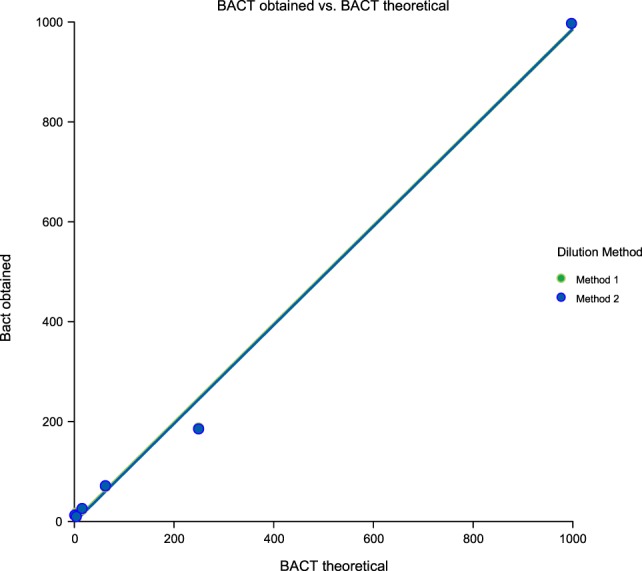
Scatter diagram of obtained and theoretical bacteria in sample 3.

Linearity results were good for WBC (*R*^2^ = 1 sample 1, *R*^2^ = 0.99 sample 2, *R*^2^ = 0.99 sample 3) (Figures 1–3), RBC (*R*^2^ = 0.99 sample 1, *R*^2^ = 1 sample 2, *R*^2^ = 0.99 sample 3) (Figures 4–6) and bacteria (*R*^2^ = 1 sample 1, *R*^2^ = 0.19 sample 2, *R*^2^ = 0.99 sample 3) on the UF-1000i (Figures 7–9). In sample 2, no linearity was found in bacteria since there was a very low initial value. Globally, it can be concluded that there is good linearity in the dilutions performed, obtaining in the UF-1000i analyser values almost identical to those expected theoretically.

#### Carry-Over

The carry-over contamination rates of RBCs, WBCs and bacteria were 0.2, 0.0 and 0.0%, respectively. The results showed that UF-1000i has practically null carry-over values.

### Screening of Significant Bacteriuria

A total of 1,220 urine specimens were included, of which 213 (17.4%) were culture positive (>10^5^ CFUs/mL urine) and 1,007 (82.6%) were culture negative. The majority of the specimens were collected from women (58.4%). The mean ages for men and women were 56.65 years (SD 23) and 44.96 years (SD 25.7), respectively. Outpatients represented 53.3% (*n* = 650) and inpatients 46.7% (*n* = 570) of the subjects. The most common microorganisms identified were *Escherichia coli* (62%), *Enterococcus faecalis* (9.4%), *Klebsiella pneumoniae* (7%), coagulase-negative *Staphylococcus* (5.6%), *Proteus mirabilis* (3.3%), *Enterobacter cloacae* (2.8%), *Pseudomonas aeruginosa* (2.8%), *Streptococcus agalactiae* (2.3%), *Citrobacter freundii* (0.9%), *Klebsiella oxytoca* (0.9%), *Streptococcus oralis* (0.9%), *Enterobacter aerogenes* (0.5%), *Enterococcus faecium* (0.5%), *Proteus vulgaris* (0.5%) and *Candida albicans* (0.5%).

Ten samples were found to be culture-positive and the Sysmex UF-1000i negative (false-negatives, 0.8%) at a cut-off value of 138.8 bacteria/μL or 119.8 leukocyte/μL. The culture results for these ten samples were: *P. mirabilis* (three); *C. albicans* (one); *P. vulgaris* (one); *E. faecalis* (one); *E. coli* (one); *P. aeruginosa* (one); *S. epidermidis* (one); and *S. agalactiae* (one).

**Figure 10:**
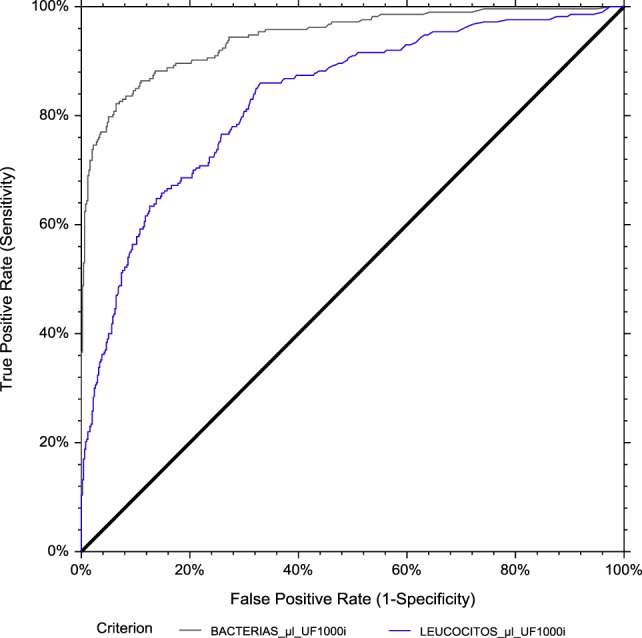
ROC curve analysis for general population. The area under the curve (AUC) is 0.943 for bacterial count and 0.832 for leukocyte count by flow cytometry, using urine culture as the reference method.

A ROC curve analysis was performed to assess the diagnostic value of bacteria and leukocyte count from flow cytometry (Figure 10). Overall, bacteria [area under the curve (AUC) 0.943] performed better than WBCs (AUC 0.832) as a predictor of culture results. For dipstick analysis, sensitivity (SE), specificity (SP), NPV, and PPV at different possible cut-off values were calculated (Table 2). The most balanced cut-off value was 89.4 bacteria/μL for bacteria, with sensitivity, specificity and NPVs of 94.8, 69.2 and 99.4%, respectively. The most balanced cut-off value was 3.8 leukocyte/μL for WBCs, with sensitivity, specificity and NPVs of 94.8, 36.7 and 97.1%, respectively.

**Table 2:** Performance of the Sysmex^®^ UF-1000i at different cut-off thresholds for leukocyte and bacteria counts.

Group		Area under the curve	Cut-off	Sensitivity (%)	Specificity (%)	Positive predictive value (%)	Negative predictive value (%)
General population	Bacteria	0.943	89.4	94.8	69.2	39.5	98.4
			255.3	90.1	79.6	48.8	97.4

	White blood cell (WBC)	0.832	3.8	94.8	36.7	24.1	97.1
			6.3	90.1	51.0	28.0	96.1

Men	Bacteria	0.956	31.3	94.9	78.3	44.6	98.8
			89.4	89.9	90.0	62.3	98.0

	WBC	0.876	3.9	94.9	44.3	23.9	97.9
			14.1	89.9	73.0	38.0	97.5

Women	Bacteria	0.946	159.3	94.8	60.9	36.0	98.1
			687.2	90.3	82.9	55.0	97.4

	WBC	0.798	3.8	94.8	32.0	24.4	96.4
			6.3	90.3	46.2	28.0	95.4

**Figure 11:**
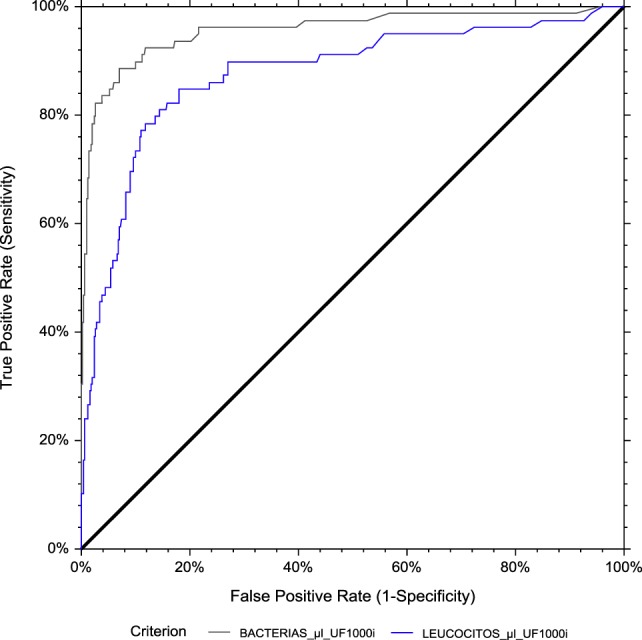
ROC curve analysis for men. The area under the curve (AUC) is 0.956 for bacterial count and 0.876 for leukocyte count by flow cytometry, using culture as the reference method.

**Figure 12:**
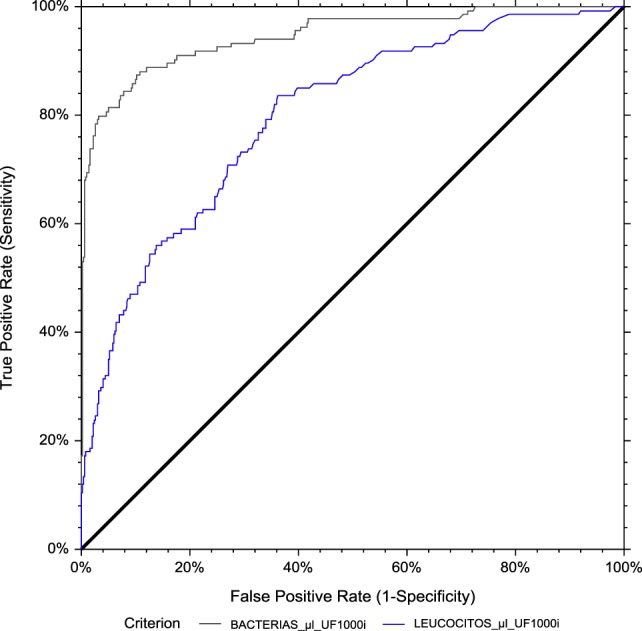
ROC curve analysis for women. The area under the curve (AUC) is 0.946 for bacterial count and 0.798 for leukocyte count by flow cytometry, using culture as the reference method.

Finally, we evaluated the effect of gender on UF-1000i performance for detecting >10^5^ CFUs/mL bacterial growth. The ROC AUCs were 0.956 and 0.946 (bacteria) and 0.876 and 0.798 (WBC) for men and women, respectively (Figures 11 and 12). The optimized cut-offs were 31.3 and 159.3 bacteria/μL and 3.9 and 3.8 WBC/μL, for men and women, respectively. Resulting SE, SP, PPV and NPV are listed in Table 2.

In the combined model using a bacteria or WBC cut-off together (138.8 bacteria/μL–119.8 leukocyte/μL), performance (SE = 95.3%, SP = 70.4%) was better than bacteria or leukocyte alone (Table 3).

**Table 3:** Performance of the Sysmex^®^ UF-1000i at cut-off for leukocyte and bacteria counts alone and in the combined model using a bacteria or white blood cell (WBC) cut-off.

Group		Area under the curve	Cut-off	Sensitivity (%)	Specificity (%)	Positive predictive value (%)	Negative predictive value (%)
General population	Bacteria	0.943	138.8	92.9	73.4	42.5	98.0
	WBC	0.832	119.8	51.2	92.5	59.2	89.2
	Bac or WBC		138.8–119.8	95.3	70.4	40.5	98.6

## Discussion

Urine is the most frequently received sample in the clinical microbiology laboratory and bacteria culture detection remains the gold standard technique for diagnosis of UTI. However, this method is time consuming and often unnecessarily applied to negative samples (3). A clinically useful screening method for UTI should be rapid, inexpensive, easy to perform and must have the highest values of sensitivity and NPV (3, 10, 16, 20). It is also important that there are no false-negatives, especially in immunosuppressed and elderly patients because of the severity of urinary sepsis. On the other hand, a high number of false positives should not be tolerated either because of the consequences of unnecessary antibiotic treatment or because of the presence of bacteria from the usual Gram-positive flora and even of over-treated bacteria that would be exposed to resistance mechanisms surviving in small clusters until the following UTI. The UF-1000i analyser has an analytic channel with specific reagent system exclusively dedicated for bacteria quantification, which has enhanced both SE and SP (21).

In this study, the results of the UF-1000i performance evaluation showed that this instrument has good precision and accuracy, and carryover was negligible. These results agree with previous studies, which have demonstrated that these systems have good precision with low interference, low carryover contamination, and are consistent with microscopic counting results (22–24). These advantages make the UF-1000i a promising screening platform for UTI (11).

We evaluated the performance of the Sysmex UF1000i in comparison with the urine culture method for screening urine samples for UTI. We established the optimum cut-off values for bacteria and leucocytes in our setting for the diagnosis of bacteriuria, assuming a cut-off >10^5^ CFU/mL as significant bacteriuria for culture test. The definition of positive urine cultures is still a matter of debate (3), De Rosa et al. (10) and Marschal et al. (25) consider 10^4^ and 10^2^ CFU/mL as significant bacteriuria, respectively. Possible false-negative cultures could be caused by the presence of dead bacteria in the urine due to treatment or a low bacterial load. In addition, we determined gender-dependent cut-off values for flow cytometric screening of urine samples.

The prevalence of UTIs in our study was 17.4% and the organisms identified were similar to those reported in literature (16, 26). The percentage of false-negatives in urine screening in this study was 0.8%, similar to that obtained by other authors (10, 22), which is around 1%. We found 10 false-negative results; three of which were *P. mirabilis* and only one was *E. faecalis*. False-negative results with Gram-negatives have been documented with the use of the Sysmex UF-1000i (10, 17, 21, 27, 28). However, other studies report false-negatives in UTIs due to *Enterococcus* spp. and *S. aureus* (21, 22, 25, 29), which should be ascribed to phenomena of aggregation of bacterial cells (14, 21).

The European Urinalysis Guidelines recommend an analytical sensitivity >90–95% to detect bacteriuria at 10^5^ CFU/mL by a rapid non-culture method with a confirmatory culture of positive cases (18, 28, 30). For a rule-out strategy, the cut-off point determination is a difficult task, as increasing test sensitivity decreases its specificity. ROC analysis is a commonly used method for determination of cut-off points at which optimal sensitivity and specificity are achieved for clinical use.

There is a great diversity of opinion in the cut-off point chosen as the most optimal to apply in the Sysmex UF-1000i system. According to the literature, it ranges from 25 to 230 bacteria/μL (10, 13, 21, 22, 29, 31). In our study, we have established an optimal cut-off point of 89.4 bacteria/μL, with sensitivity of 94.8% and specificity of 69.2% for positive cultures >10^5^ CFU/mL urine. Our results were comparable to figures reported by previous evaluation studies of the Sysmex UF-1000i. For example, Giesen et al. (32) reported cut-off values of 288.9 bacteria/μL urine or 31.8 leukocyte/μL for positive cultures >10^5^ CFU/mL urine leading to an SE of 93 and 89% and SP of 86 and 79%, respectively. In addition, March-Rosselló et al. (28) found an SE of 87.2% and SP of 85.2% with cut-off values of 247 bacteria/μL urine and SE of 70.9% and SP of73.7% with cut-off values of 31.8 leukocytes/μL urine for bacterial cultures >10^5^ CFU/mL urine. On the contrary, other studies report on significantly better results as Manoni et al. (21) that reported a cut-off value of 125 bacteria/μL urine with a sensitivity of 97% and a specificity of 94% (considering positive cultures >105 CFU/mL urine). They also reported a cut-off value of 40 leukocytes/μL urine with an 87 and 79% of SE and SP. In addition, Martín-Gutiérrez et al. (27) found an SE of 99.1% and SP of 91.5% with cut-off values of 200 bacteria/μL urine for bacterial cultures >10^5^ CFU/mL urine. The different patient population studied can explain this seemingly contradictory finding. The differences between these studies and our might be due to on divergent study designs.

There is no agreement on the usefulness of leukocyte counts as a parameter to discriminate between positive and negative urine. In some studies that took into account an improvement in operational characteristics (10, 21, 22, 28). Our results agree with these studies. In other cases, leukocyte counts did not improve the operational characteristics with respect to those obtained with only the bacterial count to discriminate between positive and negative urine, so that several authors concluded that they should not be considered in screening (14, 28, 31). The Sysmex UF-1000i and other systems such as the iQ200 classifies and quantifies the particles, including bacteria, yeasts, WBCs, and squamous epithelial cells. Nevertheless, no study has evaluated in a regressive multifactorial way the presence of epithelial cells as a NPV factor, except the study of Russcher et al. (20) comparing it with Gram staining and the *Q* index. Muñoz-Algara et al. evaluated the number of squamous epithelial cells and their relationship with contaminated urine, concluding that they can be a good predictor of contamination in urine of women of childbearing age (17). Squamous epithelial cells could, therefore, be a parameter to consider that would improve the predictive values of UF-1000i since samples considered contaminations were not excluded in our study.

For several patient groups, the general cut-off value established may not be valid, for example for pregnant women, children, immunocompromised patients, and patients on antibiotics (16). In general, female samples have higher bacterial counts than male samples, because of physiological reasons. This could partially be resolved by employing a gender-specific cut-off value (13). We have evaluated different gender-specific cut-off values, the optimized cut-offs were 31.3 and 159.3 bacteria/μL for men and women, respectively. Our results agree with the study of Jolkkonen et al. (13). These results could vary depending on the rate of negative cultures in the laboratory and the features of the population selected could influence it. Therefore, it is necessary to assess the screening method in different patient populations (20).

In our study, the sensitivity, specificity, and AUC of bacterial count in the Sysmex UF-1000i analyser system were higher than those of WBC count, and the combination of both counts for UTI screening showed sensitivity and NPV improvements to bacterial counts alone, which may help the clinical laboratory filter out true-negative samples, improving detection efficiency and reducing laboratory costs. In addition, some studies found that the NPV could be further improved when the results of WBC and bacteria were combined (15, 22). Nevertheless, some articles showed the effectiveness of screening with WBC plus bacterial counts, with an increase in sensitivity but a decrease in specificity (10, 14, 21, 26, 27).

The Sysmex UF1000i could be an interesting tool in other diseases. Grosso et al. reported a sensitivity of 84%, a specificity of 82%, and a high NPV (96%) of The UF1000i for ruling out acute non-gonococcal urethritis or predicting the presence of infection (33). In addition, the body fluid mode of this technology has been evaluated successfully to WBCs count in continuous ambulatory peritoneal dialysis, ascites fluids, cerebrospinal fluid and saliva in patients with periodontal inflammation with a sensitivity of 100, 100, 96.6 and 76%, and a specificity 86, 89, 97.4 and 78%, respectively (23, 34, 35).

This study shows that flow cytometry is a valuable method to screen urine samples to effectively rule out UTI and, may contribute to the reduction of unnecessary urine cultures. Second, the cut-offs set for the Sysmex UF-1000i in the present study allowed a reduction in culture tests. These results are important, because they allow a reduction in urine culture costs and free up laboratory resources for other activities. In addition, the cut-off values of bacteria and WBC counts depend on the study population, the type of specimens, the selected threshold for the significant count in culture, and thus must be investigated and reported by each laboratory.

## Author Contributions

AR, MM-L and MR planned and designed the experiments. MM-L, MR-A, CL and MA performed the analyses. MM-L, JG-L and AR wrote the paper.

## Conflict of Interest Statement

The authors declare that the research was conducted in the absence of any commercial or financial relationships that could be construed as a potential conflict of interest.
